# Micromachining of Invar with 784 Beams Using 1.3 ps Laser Source at 515 nm

**DOI:** 10.3390/ma13132962

**Published:** 2020-07-02

**Authors:** Petr Hauschwitz, Bohumil Stoklasa, Jiří Kuchařík, Hana Turčičová, Michael Písařík, Jan Brajer, Danijela Rostohar, Tomáš Mocek, Martin Duda, Antonio Lucianetti

**Affiliations:** 1 HiLASE Centre, Institute of Physics, Czech Academy of Sciences, Za Radnici 828, 25241 Dolni Brezany, Czech Republic; hana.turcicova@hilase.cz (H.T.); Michael.Pisarik@Hilase.cz (M.P.); jan.brajer@Hilase.cz (J.B.); danijela.rostohar@hilase.cz (D.R.); tomas.mocek@hilase.cz (T.M.); martin.duda@hilase.cz (M.D.); antonio.lucianetti@hilase.cz (A.L.); 2 Faculty of Nuclear Sciences and Physical Engineering, Czech Technical University in Prague, Brehova 7, 115 19 Prague, Czech Republic; 3 Meopta-optika, s.r.o., Kabelikova 1, 750 02 Prerov, Czech Republic; bohumil.stoklasa@meopta.com (B.S.); Jiri.Kucharik@meopta.com (J.K.)

**Keywords:** multi-beam micromachining, beam splitting, invar, shadow masks, OLED

## Abstract

To fulfil the requirements for high-resolution organic light-emitting diode (OLED) displays, precise and high-quality micrometer-scale patterns have to be fabricated inside metal shadow masks. Invar has been selected for this application due to its unique properties, especially a low coefficient of thermal expansion. In this study, a novel cost-efficient method of multi-beam micromachining of invar will be introduced. The combination of a Meopta beam splitting, focusing and monitoring module with a galvanometer scanner and HiLASE high-energy pulse laser system emitting ultrashort pulses at 515 nm allows drilling and cutting of invar foil with 784 beams at once with high precision and almost no thermal effects and heat-affected zone, thus significantly improving the throughput and efficiency.

## 1. Introduction

Invar is a Fe–Ni class alloy with unique properties such as excellent strength, impact toughness, processability and a low coefficient of thermal expansion [1]. Those properties are making invar very attractive for various industrial applications including bi-metal applications, storage tanks of liquified natural gas, and shadow masks for production of organic light-emitting diodes (OLED) [2]. High-resolution shadow masks are crucial in red, green and blue evaporation process of organic luminous materials during the production of OLED displays [3]. The geometry, size and overall quality of holes in shadow mask are directly connected with OLED pixel quality [4]. Chemical etching is nowadays a common microfabrication method for production of OLED shadow mask [2]. However, it is a complicated multi-step process generally composed of coating, cleaning, exposure and an etching process with no control over the taper angle. Moreover, it is difficult to fabricate a pattern smaller than the material thickness due to isotropic manner of chemical etching and thus reach the high resolution required for future virtual reality displays [5,6]. 

As an alternative, laser micromachining provides high-quality, single-step and environmentally friendly approach without the use of chemicals. Laser microfabrication of shadow masks was investigated with several laser systems demonstrating 85 µm channels fabricated in polymethyl methacrylate with a CO_2_ laser [7], 250 µm wide channels in wax using a CO_2_ laser [8], 250 µm wide lines in plastics and glass with the use of a Nd:YAG laser at the fundamental wavelength of 1064 nm [9], or 140 µm lines fabricated in a steel shadow mask using the third harmonics of a Nd:YAG laser [10]. However, to produce high-resolution shadow masks with small features in a range of a few tens of microns, ultrashort pulsed lasers have to be deployed [8,11,12,13]. Invar drilling with ultrashort laser systems was investigated, demonstrating 200 µm diameter in invar utilizing a 785 nm Ti:shappire laser [4] and hole dimensions below 30 µm with excellent quality and minimal heat-affected zone [6].

However, the main drawback of ultrashort pulse laser micromachining is the processing speed which limits the widespread use in industry, especially in the case of a common single-beam direct laser writing approach. Moreover, with the emerging high-power ultrashort laser systems [14], high-quality invar micromachining becomes very inefficient as most of the laser power cannot be used. This is due to the necessity of processing close to the damage threshold to maintain high quality and minimal heat affected zone. Therefore, new techniques for rapid large-scale processing are required. The most promising rapid large-scale techniques include polygonal scanning systems [15,16], direct laser interference patterning [17,18] and multi-beam scanning approaches [19].

Beam splitting using a diffractive optical element (DOE) in a combination with galvanometer scanner and high-power ultrashort laser systems is a promising way to meet industry standards in high speed processing of large areas. DOE beam-splitter distributes the incident laser beam intensity into the desired far-field pattern which is usually an 1D or 2D array of beams. Each beam has the same characteristics as the original beam, except for pulse energy and angle of propagation [20]. Multibeam systems providing high throughputs due to beam splitting into 10–100 beamlets have recently become commercially available on the market able to drill holes in the range of 20 μm in diameter into 20 μm thick metal foils [21,22].

Consequently, this study aims to demonstrate a technology capable of producing microstructures in a cost-efficient way by introducing a prototype of a Meopta beam splitting, focusing and monitoring module. The module divides an initial high-quality beam into more than 700 beamlets for parallel processing of a thin invar foil. By utilizing the module in a combination with HiLASE high-energy pulse ultrashort laser system and a galvanometer scanner, it was possible to freely adjust the dimensions and geometry of produced microholes suitable for the production of OLED displays.

## 2. Materials and Methods 

**Figure 1:**
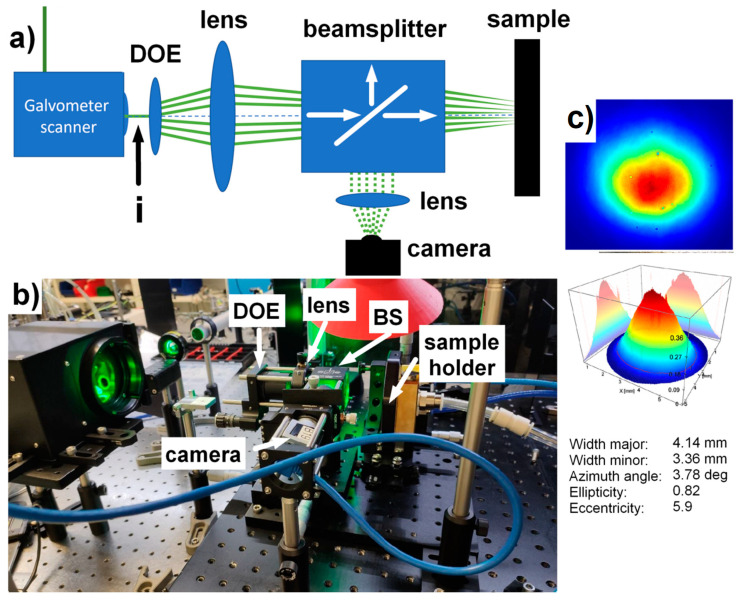
Schematics (**a**) and photo (**b**) of the experimental setup with a detail of input beam (**c**).

Invar (FeNi36) foils provided by Goodfellow GmbH (Friedberg, Germany) with a thickness of 20 µm and dimensions of 50 × 50 mm were used as received (Ra~0.07 µm). The foils were treated by Perla laser system from HiLASE (Dolni Brezany, Czech Republic), equipped with second harmonic generation module emitting at wavelength of 515 nm, with pulse duration of 1.3 ps, beam quality factor M2 better than 1.5, repetition rate of 1 kHz and pulse energy up to 10 mJ [14,23]. The generated beam (Figure 1c) was guided through the galvanometer scanner without F-theta lens into the optical module developed by Meopta-optika, s.r.o., consisting of a DOE beamsplitter, 100 mm focusing lens and a polarizing beamsplitter for pattern observation on the sample surface. The module is responsible for focusing and splitting the initial beam by means of diffractive optics into a matrix of 28 × 28 beamlets with homogeneous distribution across the whole pattern and suppression of zero order diffraction. The module allows the separation distance between beamlets to be adjusted by changing the distance between the DOE and focusing lens. Beam splitter and camera allows to monitor the intensity, shape and focal position of each beamlet. The beamsplitter passes linearly polarized beam on to the sample surface. The reflected light from the sample with random polarization is then able to reach the camera. The separation distance between each beamlet on the sample plane was 150 µm. The sample was placed vertically on to the vacuum holder with an optional connection to the cooling water circuit. The whole setup is depicted in Figure 1.

Topography of selected surfaces was analyzed with a laser scanning confocal microscope Olympus OLS 5000. The pattern shape and beamlets intensity distribution were captured by a Basler ace acA4024-29um camera with pixel size of 1.85 µm.

## 3. Results and Discussion

The impact of the experimental setup misalignments on the resulting multi-spot pattern homogeneity, the tolerance of deviation angles during beam deflections affecting the pattern shape, as well as the laser and processing parameters for high-quality cutting and drilling have been studied in a set of experiments.

**Figure 2:**
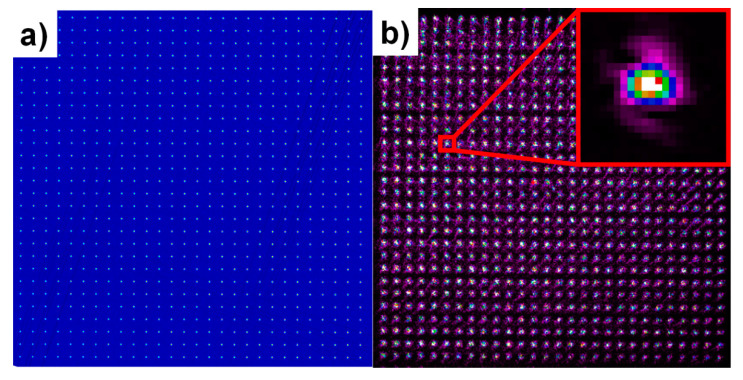
Final pattern shape on camera in a sample plane (**a**) and the same pattern reflected from the sample surface (**b**).

First, the pattern homogeneity had to be adjusted to reach the same intensity in each beamlet by tilting the beam-splitting element. The pattern shape was observed in real-time on a camera inside the Meopta beam-splitting module during the alignment, as depicted in Figure 2.

As shown in Figure 2, the pattern shape reflected from the sample surface exhibits undesired speckles, which can be further improved by mirror polishing of the sample. In addition to pattern homogeneity, the working plane was easily found by observing the pattern on camera as the sharp beam profile of each beamlet (inset of Figure 2b) can be observed only when the sample is located exactly in the focal plane. 

**Figure 3:**
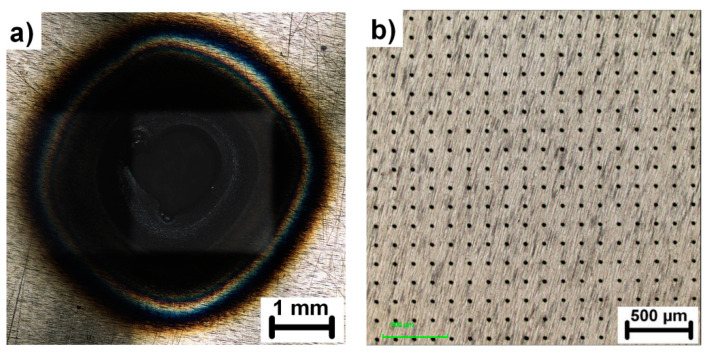
Overview of the heat affected zone on the sample processed without water-cooled holder (**a**) compared to the sample processed with the water-cooled holder (**b**). Both samples were processed with the same fluence of 0.28 J/cm^2^ and 100,000 pulses.

In the next step, the sample was irradiated with 100,000 consecutive laser pulses with energy of 0.6 mJ which corresponds to the fluence of 0.28 J/cm^2^ for each beamlet. The first experiments revealed extensive heat accumulation connected with a large heat-affected zone and interconnection of microholes into one hole in the center of the pattern due to extensive heating and melting (Figure 3a). Consequently, the samples were placed on a water-cooled holder to minimize the effect of heat accumulation, as depicted in Figure 3b.

**Figure 4:**
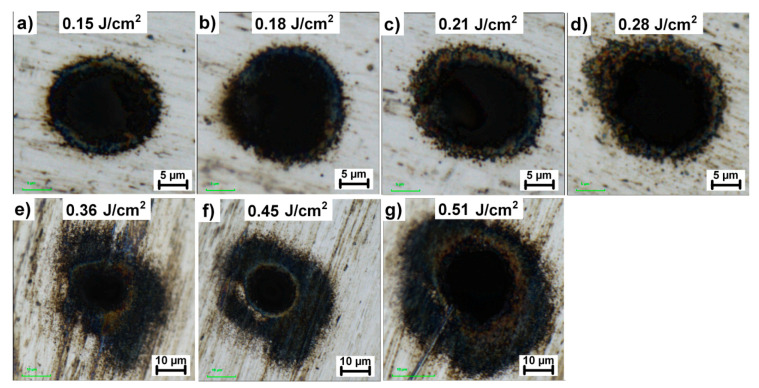
Evolution of microhole quality and heat affected zone with the increase in fluence. Sample is irradiated with 100,000 consecutive laser pulses. (**a**–**d**) gentle ablation regime, (**e**–**g**) high fluence regime.

To determine an optimal processing window for high-quality multi-beam drilling, different combinations of laser parameters were analyzed. First, the ablation threshold for the simultaneous drilling of 784 holes was estimated. Figure 4a presents a microhole ablated close to the ablation threshold with 100,000 pulses and pulse energy of 0.3 mJ in the initial beam (beamlet fluence of 0.15 J/cm^2^). No ablation was observed for pulse energies below 0.3 mJ, hence it is considered as the ablation threshold. 

The extension of the heat-affected zone and overall hole quality have been examined using different pulse energies in the range of 0.3 mJ to 1.2 mJ which correspond to the beamlet fluence of 0.15 J/cm^2^ to 0.51 J/cm^2^, as shown in Figure 4.

**Figure 5:**
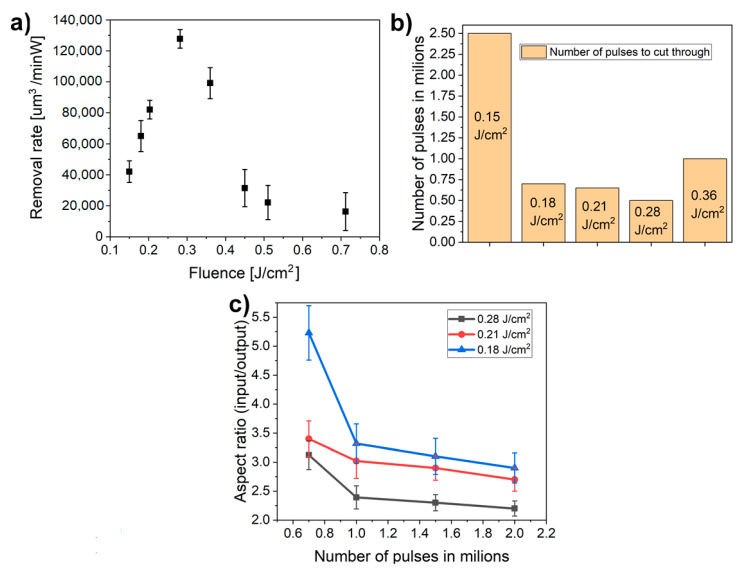
(**a**) Removal rate as a function of fluence; (**b**) Number of pulses necessary to cut through the whole sample; (**c**) Evolution of the aspect ratio as a function of number of pulses.

In line with the results presented in Figure 4, two ablation regimes have been identified. The highest quality microholes have been reached in a gentle ablation regime for fluences up to two-times the ablation threshold, i.e., up to 0.28 J/cm^2^. In this case, the extension of the heat-affected zone was found to be less than 3 µm. By increasing the fluence over this value, the quality of microholes significantly deteriorated due to the extensive heat-affected zone. Moreover, as shown in Figure 5a, the removal rate (ablated volume per time and power) for microholes drilled in a higher fluence regime over 0.28 J/cm^2^ is much smaller compared to lower fluences.

This phenomenon may be explained by the decrease in beam quality connected with high pulse energies which may overcome the tolerances of a diffractive beamsplitter and thus affect the intensity distribution within the beamlet. This issue will be further addressed in the following research.

On the other hand, the removal rate is sharply increasing with the fluence in the gentle ablation regime reaching the peak around 0.28 J/cm^2^, which was identified as the optimal fluence for effective drilling. 

Furthermore, the number of pulses required for piercing the sample have been determined (Figure 5b). With the threshold fluence of 0.15 J/cm^2^ at least 2.5 million pulses were required to pierce through the 20 µm thick invar foil, thus removing less than 0.01 nm per pulse. By increasing the fluence above the ablation threshold, the sample was pierced with less than 700,000 pulses for fluences between 0.18 J/cm^2^ and 0.28 J/cm^2^. The lowest number of pulses required for piercing the foil was in the case of 0.28 J/cm^2^ with only 500,000 pulses, which is in accordance with the optimal removal rate value. For the higher fluence regime, the sample was pierced only in the case of 0.36 J/cm^2^ after exposition with 1 million pulses. Higher fluences did not lead to cutting through the invar foil even after 3 million pulses. Therefore, the optimal processing window for effective micromachining of invar foil has been identified as 0.18 J/cm^2^ to 0.28 J/cm^2^. 

**Figure 6:**
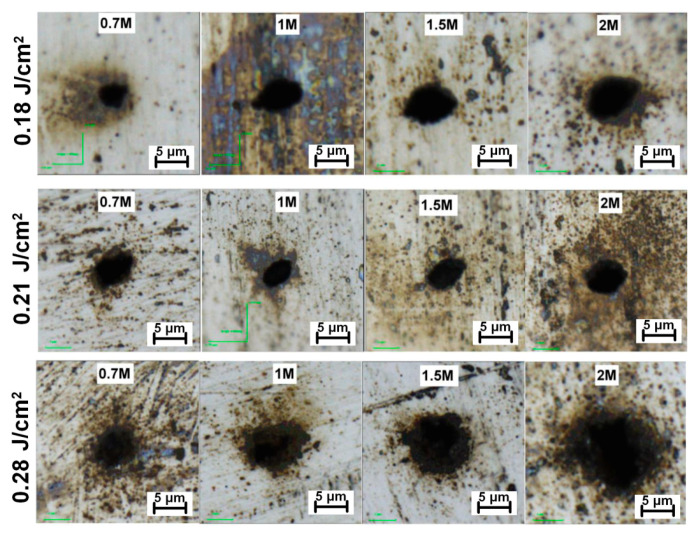
Geometry evolution of exit hole diameters with increasing number of pulses from 0.7 million to 2 million pulses.

Additionally, the effect of laser parameters on the microhole diameter input/output aspect ratio have been studied. As demonstrated in Figure 5c, the aspect ratio decreases with higher fluences as well as with increasing number of pulses. Similarly, the shape of the exit diameter also improves with a higher number of applied pulses (Figure 6).

Generally, the exit microhole diameter is larger and less elliptical for higher fluences and pulse counts, as demonstrated in Figure 6. The best aspect ratio and circularity were reached for the sample irradiated with 2 million pulses and 0.28 J/cm^2^ per beamlet.

**Figure 7:**
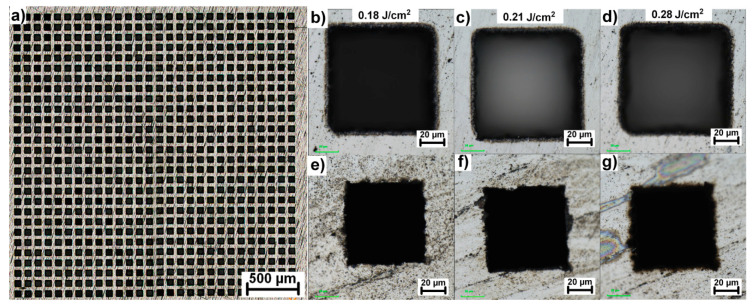
(**a**) Overview of a pattern cut with a combination of 28 × 28 beamsplitter and galvanometer scanner; (**b**–**d**) details of a square cuts fabricated with different fluences inside the optimal processing window and 400 overscans and their corresponding exit sides (**e**–**g**).

Following up on the optimal parameters, a galvanometer scanner was deployed to deflect the beam in a small area between microholes to fabricate different geometries on the invar foil. Since the separation distance between produced microholes is 150 µm, the square with a side width of 100 µm was chosen for the cutting experiment. To improve cutting efficiency, the input cut width was increased by cutting 15 squares with the same center position and decreasing diameter per 3 µm for each square. The cutting speed was adjusted to 1 mm/s to ensure high enough overlapping of ~95% with the laser repetition rate of 1 kHz. The cutting results are depicted in Figure 7.

As demonstrated in Figure 7, it was possible to fabricate square geometries and cut through the invar foil with all 784 beams at once. The complete penetration was reached for 700, 440 and 250 overscans for the fluences of 0.18 J/cm^2^, 0.21 J/cm^2^, and 0.28 J/cm^2^, respectively. In addition, the consistency of square shape and their separation distance was measured on 20 randomly selected squares across the pattern confirming the exact same parameters across the pattern with the standard deviation smaller than 3 µm in all cases.

## 4. Conclusions

Utilization of a Meopta beam-splitting, focusing and monitoring module in a combination with HiLASE high-energy pulse laser system emitting ultrashort pulses at 515 nm resulted in efficient cutting and drilling of invar foil simultaneously by 784 beamlets, arranged in a matrix of 28 × 28 beamlets. The features of optical module allow the setup to be aligned quickly to reach homogeneous intensity distribution across the whole pattern, as well as focus adjustment. By determining the ablation threshold, optimal fluence levels and pulse counts, the optimal processing window for effective high-quality drilling and cutting with heat affected zone below 3 µm has been identified as 0.18 J/cm^2^ to 0.28 J/cm^2^. The combination of galvanometer scanning head and beam-splitting module enabled multi-beam fabrication of square-shaped microholes with adjustable dimensions. With the use of this solution, the throughput can be increased 7 times compared to the state of the art commercial multi-beam systems and more than 700 times compared to the single beam approach, thus showing high potential for significant improvement in the fabrication speed and efficiency during the production of invar shadow masks.
